# Cross-sectional telephone surveys as a tool to study epidemiological factors and monitor seasonal influenza activity in Malta

**DOI:** 10.1186/s12889-021-11862-x

**Published:** 2021-10-09

**Authors:** V. Marmara, D. Marmara, P. McMenemy, A. Kleczkowski

**Affiliations:** 1 Faculty of Economics, Management & Accountancy, University of Malta, Msida, MSD 2080 Malta; 2 Faculty of Health Sciences, Mater Dei Hospital, Block A, Level 1, University of Malta, Msida, MSD 2090 Malta; 3 Department of Mathematics, University of Stirling, Stirling, FK94LA Scotland, UK; 4 Department of Mathematics and Statistics, University of Strathclyde, Rm. 1001, 26 Richmond Street, Glasgow, G1 1XH Scotland

**Keywords:** Cross-sectional surveys, Under-reporting, Seasonal influenza, Epidemiology, Influenza symptoms, Priors

## Abstract

**Background:**

Seasonal influenza has major implications for healthcare services as outbreaks often lead to high activity levels in health systems. Being able to predict when such outbreaks occur is vital. Mathematical models have extensively been used to predict epidemics of infectious diseases such as seasonal influenza and to assess effectiveness of control strategies. Availability of comprehensive and reliable datasets used to parametrize these models is limited. In this paper we combine a unique epidemiological dataset collected in Malta through General Practitioners (GPs) with a novel method using cross-sectional surveys to study seasonal influenza dynamics in Malta in 2014–2016, to include social dynamics and self-perception related to seasonal influenza.

**Methods:**

Two cross-sectional public surveys (*n* = 406 per survey) were performed by telephone across the Maltese population in 2014–15 and 2015–16 influenza seasons. Survey results were compared with incidence data (diagnosed seasonal influenza cases) collected by GPs in the same period and with Google Trends data for Malta. Information was collected on whether participants recalled their health status in past months, occurrences of influenza symptoms, hospitalisation rates due to seasonal influenza, seeking GP advice, and other medical information.

**Results:**

We demonstrate that cross-sectional surveys are a reliable alternative data source to medical records. The two surveys gave comparable results, indicating that the level of recollection among the public is high. Based on two seasons of data, the reporting rate in Malta varies between 14 and 22%. The comparison with Google Trends suggests that the online searches peak at about the same time as the maximum extent of the epidemic, but the public interest declines and returns to background level. We also found that the public intensively searched the Internet for influenza-related terms even when number of cases was low.

**Conclusions:**

Our research shows that a telephone survey is a viable way to gain deeper insight into a population’s self-perception of influenza and its symptoms and to provide another benchmark for medical statistics provided by GPs and Google Trends. The information collected can be used to improve epidemiological modelling of seasonal influenza and other infectious diseases, thus effectively contributing to public health.

**Supplementary Information:**

The online version contains supplementary material available at 10.1186/s12889-021-11862-x.

## Background

Seasonal influenza has major implications for healthcare services as outbreaks often lead to high levels of activity in the population [1] including the burden at the global level (https://www.who.int/en/news-room/fact-sheets/detail/influenza-(seasonal)). Monitoring and forecasting seasonal influenza are therefore important for health authorities and policy makers because this information support the planning of rapid interventions and appropriate control measures to mitigate its impact [2–5]. Mathematical models have been extensively used to predict epidemics of infectious diseases such as seasonal influenza and to assess the effectiveness of proposed control strategies [6–8]. However, for models to be successfully applied in a public health context, comprehensive and reliable data sets need to be available that accurately describe the incidence. This is not always the case as the reporting rate of such data can be low and they often provide just the number of cases (and not the details) about the symptoms or patient perception of the severity of the disease [1].

The issues regarding traditional surveillance data of seasonal influenza is a well-known and long-lasting discussion in public health research [2–5]. Some of these issues include: i) underreporting due to mild symptoms or limited sentinel sites; ii) severe time lags of up to 2 weeks due to the time required to collect, collate and distribute data; iii) continuous revisions of the numbers initially released as more data are recorded throughout the influenza season; iv) limited spatial resolution, typically aggregated at the country level; v) heterogeneity in the case definition across countries. To overcome some of these issues, several innovative techniques emerged to complement the traditional surveillance data and provide a better picture of the actual extent of the Influenza-Like Illness (ILI) activity in a population. Some of these methods include online surveys [9], participatory surveillance systems [2], internet traffic on specific influenza-related Wikipedia articles [10]. Some of the benefits these methods provide include the cost-effectiveness, scalability, spatio-temporal resolution, and availability in near real-time.

There are several definitions of seasonal influenza, but most include the same major symptoms. For example, the United Kingdom (UK) National Health Service (NHS) states that the symptoms related to seasonal influenza usually develop during the first 3 days upon becoming infected and include a temperature of high 38 °C or above, tiredness and weakness, headache, general aches/pains, and a dry cough [11]. All the latter symptoms are similarly defined by the World Health Organization (WHO) [12], however the WHO’s definition also includes sore throat and rhinorrhea (runny nose). The definition provided by the Centre for Disease Control and Prevention (CDC) is similar to that defined by WHO [13], with the CDC stating that it is common for children to additionally experience vomiting and diarrhoea. The definition used by the NHS has been adopted by the Health Authorities of Malta. It is unlikely that an individual will get infected more than once within the same influenza season [14], except when an individual does not develop full immunity or when a person is affected by different strains of the seasonal influenza virus [13].

Cross-sectional surveys have played a major role in many research fields such as marketing, media and political studies, but their use in surveillance of influenza is still very limited. They have been used to analyse self-perception of the illness and attitudes towards influenza vaccination [15–22]. Surveys relating to influenza can incorporate different methodologies. For example, cross-sectional serological studies are used to explore the response to immunity before and after an influenza outbreak [23], to estimate the proportion of symptomatic infected cases [24], and to estimate influenza infection rates [25]. Serological studies are also used in epidemiology to understand various characteristics related to outbreaks as well as the main predictors related to an individual’s risks in acquiring the influenza [26, 27]. For example, in a research study by Soh et al. (2012), cross-sectional serological surveys were carried out to estimate the actual infection rates of school-aged children [28]. These surveys have shown that a significant proportion of the population do not visit their General Practitioner (GP) to have their influenza-like illness (ILI) symptoms diagnosed [1, 29–31].

One of the best known recurrent seasonal influenza surveys is the UK flu survey, an online system of monitoring seasonal influenza which forms part of a European project with eleven participating countries under the project name InfluenzaNet [32]. Participants are prompted to self-report their health status on a weekly basis during the influenza season, with the aim of observing the spread of seasonal influenza through online responses regarding participants’ ILI symptoms.. The literature on InfluenzaNet data is vast and not only limited to Flusurvey in the UK. Currently, web-based participatory surveillance systems represent an important part of syndromic surveillance. Other similar examples of participatory surveillance systems are GrippeWeb in Germany [33], Flu Near You in the United States [34] and FluTracking in Australia [35]. Nonetheless, data collected through such cohort surveys have considerable bias towards those individuals that have internet access and with a higher level of education [36–38]. Despite these shortcomings, UK flu survey data are currently used by Public Health England (PHE) to monitor flu trends in the UK [32]. Flusurvey is one of the multiple data sources used by PHE (https://www.gov.uk/government/statistics/national-flu-and-covid-19-surveillance-reports). As data are available online, several researchers make use of such information. For example, Camacho et al. [39] used the data to analyse the duration of cases of ILI and acute respiratory infections (ARI) with their research findings analysed against several demographics. UK flu survey data has also been used to measure ILI and its related risk factors, suggesting that vaccination is linked to the reduced risk of becoming ill with ILI [9].

Online survey data were also used to analyse the incidence rates of seasonal influenza for different countries [33]. Similarly, in France, researchers analysed real-time data to study the spread of the seasonal influenza disease [38]. In Spain, other researchers made use of their data to compare the incidence rates of countries that are participating in this project [33]. In addition, an online tool for self-reporting of ILI is being used to understand the mechanisms of the spread of seasonal influenza in Denmark [40].

Tan et al. (2013) found that surveys provided useful information about key epidemiological parameters in relation to seasonal influenza [24]. Surveys have also been used to obtain improved and more informative prior distributions [41]. Such prior information can help mathematical models to obtain better forecasts when predicting influenza outbreaks. However, limited research exists about the application of nationwide cross-sectional survey data to improve the understanding of the prior distributions of seasonal influenza outbreaks. Telephone surveys (as used in this study) can offer a good solution to fill missing gaps about knowledge related to the seasonal influenza cases [34].

One of the key limitations for the use of models to describe and predict outbreaks of infectious diseases like seasonal influenza is a gap between the number of actual cases and what can be and is reported [1]. The under-reporting rates – as estimated by serological studies – can be very severe, with only a small proportion of actual cases reported to the authorities.

In this study, we introduce a novel method utilising telephone surveys which, to our knowledge, has not yet been used in the context of monitoring infectious disease outbreaks in Malta. We use telephone surveys to gain additional information about seasonal influenza outbreaks in Malta during the 2014–15 and 2015–16 seasonal influenza epidemics. This information is then compared to the GPs data as well as Google Trends data. We also compare the results of the surveys to other sources of epidemiological data for seasonal influenza epidemics in Malta in two seasons: 2014–15 and 2015–16.

## Methods

For the scope of this paper, a distinction is being made for the terms “seasonal influenza”, “ILI” and specific symptoms, which include the following:

1. GP diagnosed ILI cases refer to those patients who were diagnosed with the illness by GPs;
2. Self-reported influenza cases are those cases reported by the individuals themselves and who responded to the surveys;
3. Self-reported ILI cases refer to those individuals who self-reported at least one specific symptom related to seasonal influenza as defined above by NHS and WHO [3–5].
4. With reference to ‘seasonal influenza’ cases throughout the paper, this reflects the estimated number of the seasonal influenza cases and not the actual reported seasonal influenza cases. The actual seasonal influenza cases can only be recorded through serological tests, which at the time of study, were unavailable in Malta.

### Cross-sectional surveys

The influenza season in Malta varies approximately between October and May and usually peaks between January and February. Two cross-sectional surveys were carried out at two different time points (across 2 years). Since the same questionnaire was used for both surveys, Survey 1 and Survey 2 shall refer to the ‘first wave’ and the ‘second wave’ of this national study. Survey 1 was carried out between week 35 (end of August 2015) and week 37 (September 2015), and its primary aim was to explore the under-reporting rate of seasonal influenza as compared with the data collected by the GPs during the 2014–2015 seasonal influenza. The questionnaire (Supplementary File 1) consisted of 32 questions related to whether participants had experienced seasonal influenza, whether they had any specific symptoms, and included queries on socio-demographic factors. Furthermore, respondents were given a list of symptoms to evaluate, and were asked whether they had experienced any of these symptoms as a single symptom or a group of symptoms during the same instance or at multiple times during the past year. Following this list, respondents were asked to indicate the months in which they experienced these symptoms. Through these responses, a time series curve was developed for the above two influenza seasons individually (‘symptomatic cases per month’). Similarly, respondents were asked to indicate the months in which they experienced the ‘seasonal influenza’ and hence another time series curve was also constructed for this variable (‘influenza cases per month’). The full questionnaire is available upon request from the corresponding author.

This study was preceded by a pilot survey with a small random sample of 20 individuals to ensure that all questions were clear and comprehensible, as well as to ascertain the practicalities of conducting the telephone survey. The initial results through the pilot study showed that the tool was coherent and could be conducted via telephone, therefore no further changes were made. The respondents from this pilot study were not included in the larger study.

The second survey (Survey 2), using the same questionnaire as Study 1, was carried out between week 17 (end of April 2016) and week 19 (May 2016) of the 2015–16 influenza season. Thus, the fact that the second survey was carried out between weeks 17–19 as compared to weeks 35–37 in Survey 1 may have resulted in lower recall bias as respondents might have found it easier to recall their ILI symptoms. The different timings allow us to analyse the reliability and consistency of surveys, and the implications of the timing of the survey for the results.

The interviews were conducted in Maltese but if participants preferred to answer in English, this option was offered at the start of the survey. In each wave of the survey, a random sample of 406 Maltese individuals was recruited from the eligible population of residents of Malta (349,724 individuals) aged 18 and over. The study was carried out using a 95% confidence level with a maximum margin of error +/− 4.86% using a sample size calculator.^1^ Telephone numbers were generated from a random number generator (Microsoft Excel). To reach the desired sample, a total of 720 individuals were contacted for Survey 1 and 406 responded to the survey (with 56% response rate); a total of 698 individuals were contacted for Survey 2 to reach the same sample size (with 58% response rate). To ensure representativeness of the population, both samples were stratified based on demographical data: sex, district and age. The population proportions for these demographics were obtained from the National Statistics Office (NSO) of Malta [42].

### General practitioners’ (GPs) data

The survey results were compared to GP consultations data regarding ILI, which were collected by the Malta Health Promotion Department (MHPD) for two influenza seasons: 2014–15 and 2015–16. The average number of GPs reporting the weekly ILI cases for both influenza seasons was 5.7 GPs. For the 2014–2015 influenza season, data are available between week 40 and week 21, while for the subsequent season between week 41 and week 20.

Traditional surveillance data cover all patients visited by the sentinel GPs, including those diagnosed with ILI, thus allowing estimation of the number of ILI cases in the overall population. The Maltese Health Authorities classify a positive ILI case when a person has a sudden acute illness with several symptoms as classified by the NHS (onset during the last 7 days), with measured temperatures of > 38 °C. The number of ILI diagnosed individuals was estimated to be 31,514 during the 2014–15 season, and 29,090 during the 2015–16 season. This was scaled up to the population of Malta, considering that there were 300 GPs practicing at that time. Hence, the reported numbers by the MHPD (by an average of 5.7 GPs) were scaled up to 300 GPs.

### Google trends

Google Trends provides data that represent the search interest relative to the highest point on the chart for the given region and time. A value of 100 is the peak popularity for a particular term, with a value of 50 indicating that the term is half as popular. Likewise, a score of 0 means the term was less than 1% as popular as the peak”.^2^ Google Trends was used to obtain time series data for the two influenza seasons studied here (Season 1: September 2014 – August 2015 and Season 2: September 2015 – August 2016). Key word searches such as ‘Influenza’, ‘Flu’ and ‘Cold’ in Maltese and English languages that originated in Malta were used to generate the time series data. However, it does not give the actual number of times that the search was carried out. As a result, the relative number of searches for influenza-based terms each season is unknown. Since the search was carried out on six keywords, which are all related in some way to the seasonal influenza, one time series curve for each season was obtained. The weighting for each keyword was based upon the number of keyword searches with more searches equating to higher weightings. For both seasons (2014–15 and 2015–16), January was the month with the highest proportion of searches related to influenza.

### Comparison between different data streams

We use the following proxy measures for the number of seasonal influenza cases, as discussed in this section:

1. Respondents that self-reported seasonal influenza (survey data);
2. Respondents who self-reported seasonal influenza cases in households (survey data);
3. Respondents that self-reported ILI symptoms (survey data);
4. Respondents that reported high fever (survey data);
5. Diagnosed ILI cases reported by the GPs;
6. Google Trends records.

We translated the surveys’ results into a proxy measure of the number of cases by first calculating the proportion of positive answers to the relevant questions and subsequently multiplying the proportion by the size of the Malta population. We are aware that differential susceptibility exists for seasonal influenza among different age groups [21]; however due to the lack of demographical data in relation to seasonal influenza gathered by Health Authorities in Malta, the results were also assumed to be representative of the whole population of Malta.

### Data analysis

Statistical analyses were performed using SPSS® version 21.0. Descriptive and inferential statistics, such as percentages, frequencies, means, standard deviations and confidence intervals, were used to present the basic statistics in relation to the demographics, general medical information variables and other information related to the seasonal influenza. The confidence intervals for the estimates of the seasonal influenza cases were based on the respective margin of error (±4.86%) of the cross-sectional surveys. Chi-square tests were used to assess differences between categorical variables, and Pearson’s correlation coefficient was used to assess the association between continuous variables. Statistical significance was established at *p* < 0.05 for all analyses. No adjustment was made for multiple testing.

### Ethical considerations

Ethics approval was obtained from the Psychology Ethics Committee at University of Stirling (28th August 2015). Following an explanation of the main purpose of this research to the participants, individuals were invited to participate in the study through a telephone survey. Participants were given the option to opt out from this research study at any time during the 5-min telephone survey. Furthermore, respondents were also assured that all the collected information would be processed anonymously and confidentially. Once respondents agreed to participate in survey, none opted out during the data collection.

## Results

Results are presented in three parts. Firstly, we present general features of the surveyed population, such as demographic structure, medical information, and willingness to accept influenza vaccine. Secondly, we concentrate on survey results directly corresponding to ILIs, i.e., self-reporting of individual or household symptoms. Thirdly, we use these results as indicators of the influenza prevalence, comparing the results with the data obtained from sentinel GPs and Google Trends.

### General features

#### Population profile

The participant details for the two surveys are given in Table 1. Both samples have very similar sample characteristics, and any differences in the sample characteristics between both surveys are within the margin of error of ±4.86%. The demographic variables ‘Sex’ and ‘Age’ are comparable with official Maltese statistics at the time when the surveys were carried out.

**Table 1:** Basic sample demographics for Surveys 1 and 2

	***n*** = 406	Survey 1	***n*** = 406	Survey 2	Population Data [32]
***Sex***					
Female	207	51.0%	212	52.2%	50.5%
Male	199	49.0%	194	47.8%	49.5%
***Current Status***					
Employees	189	46.5%	183	45.1%	n/a
Pensioners	89	21.8%	93	22.8%	n/a
Housewives/husbands	87	21.5%	91	22.5%	n/a
Students	30	7.5%	33	8.1%	n/a
Unemployed	11	2.7%	6	1.5%	n/a
***Level of Education***					
Primary	76	18.7%	79	19.5%	n/a
Secondary	222	54.6%	212	52.3%	n/a
Diploma	55	13.5%	60	14.9%	n/a
Tertiary	54	13.2%	54	13.3%	n/a
***Age***					
18–25	53	13.1%	49	12.1%	12.5%
26–35	58	14.3%	64	15.8%	18.3%
36–45	63	15.5%	55	13.6%	15.2%
46–55	71	17.5%	63	15.5%	17.8%
56–65	91	22.4%	82	20.2%	17.8%
66+	70	17.2%	93	22.9%	18.4%
			**Average number of individuals per household**		
		2.9 (SD = 1.1)		2.8 (SD = 1.0)	

#### Participants’ general medical information

We found that on average, the participants reported visiting their GP 2.7 times (Survey 1 SD = 2.25; Survey 2 SD = 4.99) in 1 year (for both surveys), with the majority visiting their GP twice a year (26.4%, 107/406), followed by once a year (18.5%, 75/406) and three times a year (16.3%, 66/406), with 38.8% visiting more than three times a year. These visits were related to all health problems, not necessarily seasonal influenza. We also found that 41.2% of the participants (167/405) took regular medication due to medical conditions such as asthma, diabetes or heart disorders. In the older age group (66+) (89.9%, 63/70), the proportion taking regular medication was significantly higher when compared to the younger generation (χ^2^ (5) = 121.11, *p*-value < 0.01). For those between the age of 18 and 25 years, 17.0% (9/53) reported taking regular medication; for those between 26 and 35 years, 8.6% (5/58) took regular medication; for those between 36 and 45 years, 22.2% (14/63) took regular medication, and for those between 46 and 55, 39.4% (28/71) took regular medication. Furthermore, results exceed the 50% threshold for the age group 56–65 (53.8%, 49/91). These results are identical between both surveys.

#### Willingness to accept influenza vaccine

The Maltese Government offers the seasonal influenza vaccine free of charge to some groups of individuals (healthcare professionals, young children, elderly people, chronic disease patients and other employees). Everyone else needs to consult their GP to receive their seasonal influenza vaccination at a cost. According to Survey 1 results, 43.1% (175/406) reported that they had received the flu vaccine, 55.4% (225/406) had not taken the vaccine, and 1.7% (7/406) did not remember. Of those who received the vaccine, the 66+ age group (73.9%, 51/69) was the only age group that exceeded 50% uptake. We found that there is a significant association between the different age groups when compared with the vaccine uptake (χ^2^ (10) = 49.86, *p*-value < 0.01). These results are similar in both surveys.

The individuals who did not take the seasonal influenza vaccine responded as: ‘not interested’ (41.1% of individuals, 92/224), followed by those who were afraid (24.1%, 54/224), and 10.7% (24/224) who said that they ‘feel sick after taking the vaccine’. Similar reasons were provided for Survey 2.

Thus, a significant association was found between the different age groups and the vaccine uptake. This result is due to the Maltese Government’s inclusion criteria for the free vaccine. Furthermore, the latter result is similar to England’s seasonal influenza vaccine uptake rate for those aged 66+ [43, 44]. Those between 18 and 25 years of age are the least likely age group to take the seasonal influenza vaccine (22.6%, 12/53), while those between 26 and 65 years the uptake rate varied between 36 and 46%.

### Self-reporting

#### Seasonal influenza reporting

In this section, we carry out analysis on responses to questions directly related to the term ‘seasonal influenza’. The respondents were asked whether they had seasonal influenza during the past year, without revealing the standard definition. Hence, results here are based on their own understanding of the symptoms of seasonal influenza, and/or on their GP’s advice. Results from Survey 1 show that 29.8% of the individuals (121/406) stated that they had seasonal influenza in the period while for Survey 2, 37.2% (151/406) reported having seasonal influenza. Furthermore, 67.0% of respondents in Survey 1 (272/406) claimed that they did not acquire seasonal influenza (62.8%, 255/406 - Survey 2) and 3.2%, 13/406 were unsure (0% - Survey 2). The most common month for the seasonal influenza according to the respondents in both surveys was January, followed by February and December (Table 2).

**Table 2:** Months in which participants indicated having the seasonal influenza (Respondents were able to indicate more than 1 month)

	***n*** = 121	Survey 1%	***n*** = 151	Survey 2%
**August**	0	0.0%	0	0.0%
**September**	0	0.0%	1	0.6%
**October**	15	8.2%	1	0.6%
**November**	11	6.0%	14	7.5%
**December**	30	16.4%	32	17.3%
**January**	52	28.4%	65	34.7%
**February**	42	23.0%	57	30.6%
**March**	26	14.2%	16	8.7%
**April**	4	2.2%		n/a
**May**	2	1.1%		n/a
**June**	1	0.5%		n/a
**July**	0	0.0%		n/a
**Total**		100.0%		100.0%

We also asked people for the duration of their seasonal influenza. The respondents claimed that on average the duration of their seasonal influenza was 9.9 days (SD = 7.22) (Survey 1) and 9.5 days (SD = 3.84) (Survey 2). This is similar to a generally accepted recovery period 1 week, with a complete recovery taking up to 10 days [4, 34, 45].

Respondents could report more than one instance of having the seasonal influenza. Those who stated they had seasonal influenza also claimed to have contracted seasonal influenza an average of 1.50 times (Survey 1: 54.2% experienced it once, 41.5% experienced it twice, 4.2% experienced it three times) and 1.28 times (Survey 2: 72.8% experienced it once, 27.2% experienced it twice) during the year. There can be several reasons for this effect. Some people with a lower immune system might suffer from seasonal influenza more than once [13], or might suffer from influenza A (which is the common strain of seasonal influenza) and influenza B [45, 46]. However, most people reporting seasonal influenza more than once in a year might have misinterpreted their ILI symptoms as another case of seasonal influenza.

### Infection of household members

Most respondents in Survey 1 (54.4%, 221/406) claimed that at least one additional member from their household had acquired seasonal influenza. On the other hand, many respondents in Survey 2 did not claim that at least one additional member from their household had acquired seasonal influenza (24.9% 101/406). On average, from every household, 1.8 household members (SD = 0.95) acquired the seasonal influenza (Survey 2–1.7 members, SD = 0.97). However, when also taking into account those who claimed (telephone respondents) that they had seasonal influenza, 61.1% of the households included in the study (248/406) had at least one person with seasonal influenza (43.8%, 178/406 - Survey 2).

### Symptoms

#### General symptoms

In this part of the study, respondents were asked whether they had experienced specific symptoms from a list of ILI symptoms such as fever, cough, sore throat, headaches and other symptoms. However, the survey here was not limited to those who self-reported having ILI, thus allowing us to estimate the general prevalence of symptoms. Specific symptoms were individually stated to respondents, allowing the respondents to select each applicable one. Respondents were asked to reply to this question retrospectively for the period August 2014 – July 2015 in Survey 1 (carried out in August/September 2015) and August 2015 – March 2016 in Survey 2 (carried out in April/May 2016). Table 3 provides the percentages based on the total number of symptoms mentioned for both surveys.

**Table 3:** Individual results for 16 ILI symptoms

	***n*** = 406	Survey 1%	***n*** = 406	Survey 2%
			Symptoms	
**Runny or blocked nose**	250	61.6%	237	58.4%
**Headache**	246	60.6%	227	55.9%
**Sore throat**	221	54.4%	205	50.5%
**Cough**	203	50.0%	198	48.8%
**Sneezing**	186	45.8%	222	54.7%
**Feeling tired or exhausted**	167	41.1%	125	30.8%
**Muscle/joint pain**	141	34.7%	140	34.5%
**Fever**	116	28.6%	103	25.4%
**Loss of appetite**	91	22.4%	47	11.6%
**Watery eyes**	86	21.2%	106	26.1%
**Diarrhoea**	73	18.0%	65	16.0%
**Shortness of breath**	68	16.7%	63	15.5%
**Stomach ache**	61	15.0%	49	12.1%
**Nausea**	54	13.3%	37	9.1%
**Chest pain**	47	11.6%	49	12.1%
**Vomiting**	28	6.9%	16	3.9%

The above results are sorted in descending order to elicit the most common symptoms amongst the participants from both surveys. Respondents were asked to reply for each symptom

The most commonly reported symptoms were ‘runny or blocked nose’, followed by headache, whilst the least common symptoms were vomiting and chest pain (Table 3). According to Survey 1, 15.5% of the Maltese population (63/406) did not suffer from any of the above symptoms during the indicated period (20.0%, 81/406 - Survey 2). For Survey 2, a higher number of individuals did not experience any of the above symptoms; however, the second survey was based on a shorter time period (since Survey 2 was carried out in April, while Survey 1 in August).

In Table 3 we represent symptoms that could be associated with seasonal influenza. Hence, an individual might not self-report having had the seasonal influenza, but nevertheless could have had it. Hence, the 84.5%, 343/406 (80.0%, 325/406 - Survey 2) who claimed to have any of these symptoms are the maximum boundary number of individuals that might have had the seasonal influenza.

#### Symptoms for those who self-reported

Another approach we took was to limit the analysis of symptoms to those participants who self-reported having seasonal influenza. Thus, we are looking here at what symptoms people associate with the ILI, rather than those that they might have experienced in general. In Table 4 we order the symptoms by what proportion of patients reported a particular one. On average, respondents suffered 5.4 symptoms in Survey 1 and 7.2 symptoms in Survey 2. All the individuals who indicated that they had seasonal influenza mentioned at least one symptom. Similar to Table 3, most participants reported cough, sore throat and fever whereas the least frequently mentioned symptoms were watery eyes, vomiting and nausea.

**Table 4:** A comparison (Survey 1 vs. Survey 2) between the symptoms related to the seasonal influenza, as mentioned by the survey respondents

Symptoms	n	Survey 1%	n	Survey 2%	Difference
**Cough**	102	15.5%	142	13.1%	−2.4%
**Sore throat**	93	14.1%	141	13.1%	−1.1%
**Fever**	80	12.2%	84	7.8%	−4.4%
**Runny or blocked nose**	70	10.6%	138	12.8%	2.1%
**Headache**	70	10.6%	101	9.4%	−1.3%
**Sneezing**	58	8.8%	143	13.2%	4.4%
**Muscle/joint pain**	**35**	**5.3%**	**126**	**11.7%**	**6.3%**
**Feeling tired or exhausted**	31	4.7%	46	4.3%	−0.5%
**Stomach ache**	22	3.3%	5	0.5%	−2.9%
**Diarrhoea**	20	3.0%	29	2.7%	−0.4%
**Shortness of breath**	18	2.7%	21	1.9%	− 0.8%
**Loss of appetite**	18	2.7%	19	1.8%	−1.0%
**Chest pain**	17	2.6%	26	2.4%	−0.2%
**Nausea**	10	1.5%	12	1.1%	−0.4%
**Vomiting**	10	1.5%	2	0.2%	−1.3%
**Watery eyes**	4	0.6%	45	4.2%	3.6%
**Total**		100.0%		100.0%	

Please note respondents were allowed to mention more than one symptom

Responses between both surveys were similar, with the exception of the ‘Muscle/joint pain’ symptom where more participants reported it in Survey 2 than 1 (+ 6.3% difference). This again demonstrates consistency of the survey method across different seasons.

Although the results in Table 3 and Table 4 are similar, they represent different ways in which respondents perceived the ILI symptoms. Thus, only some of them (cough, sore throat, fever) are clearly associated in people’s memory with the seasonal influenza, other symptoms, like running nose or sneezing, are more generic.

#### Timing of influenza-like illness (ILI) symptoms

The participants also were asked to identify the months in which they experienced ILI symptoms, see Table 5. As reported in Survey 1, the most common month for the above symptoms was January 2015, followed by February 2015 and March 2015. The least common months were August 2014, September 2014 and July 2015 (Table 5). The second survey was carried out earlier (April) when compared to Survey 1 (August), hence respondents for Survey 2 were not able to report their status in the period from April to July. However, in Survey 2, January to March were still the most popular months with the above symptoms.

**Table 5:** Months where participants indicated as having any of the symptoms, listed in the questionnaire

	***n*** = 343	Survey 1%	***n*** = 325	Survey 2%
**August**	2	0.3%	0	0.0%
**September**	2	0.3%	12	2.3%
**October**	38	5.8%	11	2.1%
**November**	41	6.3%	38	7.3%
**December**	70	10.7%	76	14.6%
**January**	123	18.8%	137	26.2%
**February**	104	15.9%	154	29.5%
**March**	95	14.5%	94	18.0%
**April**	70	10.7%		n/a
**May**	48	7.3%		n/a
**June**	43	6.6%		n/a
**July**	18	2.8%		n/a
**Total**		100.0%		100.0%

Please note respondents were able to indicate more than 1 month. The second survey was carried out earlier (April) when compared to Survey 1 (August), hence respondents for Survey 2 were not able to mention from April to July months

On average, the respondents reported that their symptoms persisted for 9.4 days (SD = 7.72) in Survey 1 and 5.9 days (SD = 3.97) in Survey 2. The difference between the average number of days for Survey 1 and Survey 2 shows that different seasons might have different characteristics related to seasonal influenza or its symptoms of reflect different timing of surveys in relation to outbreaks. For people with the symptoms, listed in Tables 3, 56.5% (194/343 - Survey 1) and 55.7% (181/325 - Survey 2) claimed that they were restricted to stay at home to recover from their ILI symptoms.

#### High temperature

Respondents were also specifically asked if they had suffered from high body temperature. Out of the individuals who reported having seasonal influenza (including those who opted for the ‘don’t know’ option), 64.5% (78/121) claimed that they had high temperature in Survey 1, while 55.0% (83/151) in Survey 2, 22.3% (27/121) (42.4%, 64/151 - Survey 2) did not and 13.2% (16/121) (2.6%, 4/151 - Survey 2) did not know. Furthermore, 68.6% (83/121) visited a doctor due to their seasonal influenza (72.8%, 110/151 - Survey 2), 19.0% (23/121) did not (27.2%, 41/151 - Survey 2) and 12.4% (15/121) did not remember (0% - Survey 2). We also found that four out of every five respondents claiming to have contracted seasonal influenza took medicine to cure their symptoms (97.4%, 147/151- Survey 2), while 13.2% (16/121) did not remember (0% - Survey 2). On the other hand, 19.8% of respondents in Survey 1 (24/121) were hospitalised due to the seasonal influenza with only 4.0% in Survey 2 (6/151). The hospitalised individuals spent an average of 6 nights (SD = 5.16) (Survey 1) and 7.7 nights (SD = 1.82) (Survey 2) at hospital.

#### Comparing different data

In this section, we use the survey results as proxies for estimating the actual number of cases and compare these numbers with the results obtained from other sources, the GP reports and Google Trends. We first look at the total incidence and subsequently compare the time dependence in different streams of data.

#### Self-reporting

Based on the results of Survey 1, the baseline total number of seasonal influenza cases in Malta between October 2014 and Mid-May 2015 can be estimated as ca. 130,000 cases. This number is obtained by multiplying the proportion of respondents in Survey 1 who reported having seasonal influenza (29.8%) by the total population of 425,384 [42]. According to the data obtained from the Health Authority, an estimate of 32,000 seasonal influenza cases were reported by GPs in the same period. Based on this calculation, this implies that the official statistics provided by GPs is close to 25% reporting rate. After applying a similar calculation using Survey 2 data, this would result in a reporting rate equal to 18.1% (based on 37.2% of respondents who reported having seasonal influenza), thus stressing consistency between both surveys (Table 6). However, this number likely underestimates the incidence, as people might have experienced seasonal influenza more than once. Using the 1.5 multiplier that we obtained from Survey 1 as the number of times the respondents felt they had seasonal influenza within a year, we obtain 195,000 cases (Table 6) and a reporting rate for the official records of 16.4% (14.5% for Survey 2).

**Table 6:** Number of cases for the two seasonal influenza seasons (2014/2015 and 2015/2016) as self-reported by survey respondents based on 4 different variables

	2014–15 (Survey 1) Number of influenza cases	2015–16 (Survey 2) Number of influenza cases
**Seasonal influenza cases**	195,000 (CI: 185,000 – 205,000)	200,000 (CI: 190,000 – 210,000)
**Symptomatic cases**	360,000 (CI: 342,000 - 378,000)	340,000 (CI: 324,000 – 357,000)
**Individual’s temperature**	180,000 (CI: 171,000 - 189,000)	140,000 (CI: 133,000 – 147,800)
**Influenza cases in households**	230,000 (CI: 218,800 - 242,000)	135,000 (CI: 128,000 – 142,000)

#### Cases in households

According to Survey 1 results, 61.1% of all households in Malta had at least one household member with seasonal influenza, with an average of 1.8 people per household experiencing the seasonal influenza. According to the Maltese National Statistics Office (NSO), the total number of households in Malta is around 140,000 [47]. By using the latter data and considering that an individual might have experienced the seasonal influenza 1.5 times (Survey 1) during the same season, we can estimate that there were around 230,000 seasonal influenza cases (Table 6) during the 2014–15 season. Therefore, based on the GPs data, this result indicates that the reporting rate for the GP official records is 13.9%. When applying the same methodology to the 2015–16 dataset, the reporting rate is 21.5%.

#### Number of respondents reporting at least one symptom

Based on the results of Survey 1, the baseline total number of seasonal influenza cases in Malta between October 2014 and Mid-May 2015 can be estimated as ca. 360,000 cases. This number is obtained by multiplying the proportion of respondents in Survey 1 who reported having at least one symptom for seasonal influenza (84.6%) by the total population of 425,384 [42]. Based on this calculation, this implies that the official statistics provided by GPs are as low as 9%. After applying a similar calculation using Survey 2 data, this would result in a reporting rate equal to 8.5% (based on 80% of respondents who reported having at least one seasonal influenza symptom), again showing consistency between both surveys (Table 6). However, this is a clear overestimation of the total number of cases as we are assuming that all ILI symptomatic individuals eventually acquired the seasonal influenza, which is highly unlikely.

#### Individuals’ temperature

One of the most significant symptoms of seasonal influenza is fever [45]. From all respondents, 28.6% claimed to have experienced fever during the year. If we consider this percentage as an estimate of the number of seasonal influenza cases, and again consider that individuals might have contracted seasonal influenza on average 1.5 times per year (as reported in Survey 1), we obtain a total of 180,000 seasonal influenza cases (Table 6) during 2014–15. This result provides a reporting rate of 17.8%. The same calculations carried out for the 2015–16 survey yield a reporting rate of 20.7%.

#### Time dependence

**Fig. 1:**
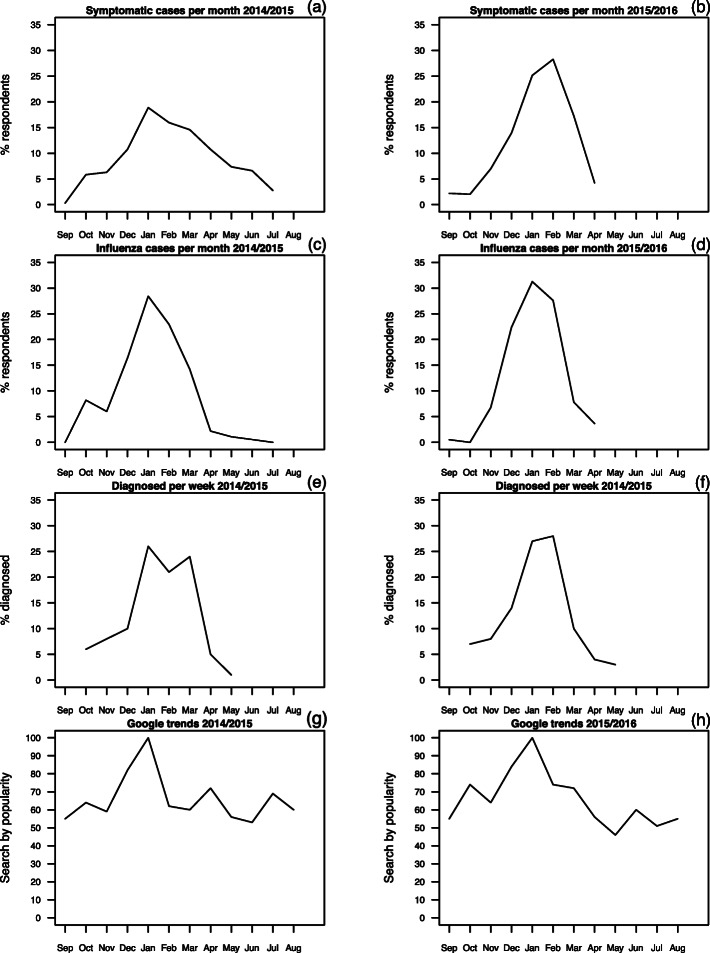
**a** % of the monthly occurrences of the 2014–15 Influenza-like Illness (ILI) symptomatic cases as stated by the survey respondents from the total monthly occurrences mentioned, (**c**) % of the monthly occurrences of the 2014–15 seasonal influenza cases as stated by the survey respondents from the total monthly occurrences mentioned, (**e**) % of the weekly GP diagnosed ILI cases for the 2014–15 season from the total number of diagnosed ILIs throughout the whole season, (**g**) search by popularity of the Google search trends for the 2014–15 season using the key words ‘Influenza’, ‘Flu’ and ‘Cold’ in both Maltese and English language for searches that were carried out in Malta. Charts (**b**), (**d**), (**f**) and (**h**) have the same definitions as (**a**), (**c**), (**e**) and (**g**) respectively but for the 2015–16 season

Data gathered from the surveys shed more light on different characteristics of seasonal influenza such as symptoms, months in which respondents claimed to have several ILI symptoms, and months when they thought they acquired the seasonal influenza. The latter two variables can be directly compared with the data obtained by a more traditional route of GP reporting (Fig. 1).

There is a good agreement between the shape of the time dependence of monthly reports of the ILI symptomatic cases (as stated by the survey respondents) (Fig. 1a) and the 2014–15 diagnosed ILI cases (GPs reported data) (Fig. 1e). This agreement is reflected by a strong linear correlation (*r =* 0.90; *p*-value = 0.002). Although the survey data were collected retrospectively, the respondents appear to remember the actual months when they had the symptoms. Interestingly, in May, the survey data registered a higher number of symptoms reported in Survey 1, when compared with the observed diagnosed ILI data (Fig. 1a).

Similarly, in Fig. 1c, the monthly reports of seasonal influenza cases, as stated by the survey respondents, show good agreement with the seasonal influenza cases as reported by the GPs (Fig. 1e), with high correlation (*r* = 0.88, *p*-value = 0.004). The data for the last 3 months show lower values than the one based on the symptoms (compare Fig. 1a and c).

The two survey variables, symptoms and self-diagnosis, are also correlated (*r* = 0.85, *p* = 0.008). However, only around 30% of participants claimed they had seasonal influenza, while around 84.6% claimed that they had any of the above ILI symptoms. Hence, based on these results, it is likely that respondents may have a different perception of the definition of seasonal influenza or the difference is in the survey design since some of these symptoms overlap with other illnesses. Therefore, illness perceptions and health beliefs can be rather subjective, although are important predictors for health utilization [17, 18].

Similar results were obtained for the 2015–16 survey (Fig. 1b, d and f and Table 7). The months in which the respondents reported the symptoms or self-diagnosed seasonal influenza are comparable to the months that were associated with high levels of ILI cases as recorded by the GPs (compare Fig. 1b and d with f).

**Table 7:** Correlation analysis for the three variables related to the months of the influenza symptoms (2015–16 season). ‘GPs_Influenza’ is the diagnosed seasonal influenza individuals collected by the GPs, while ‘Survey_Symptoms’ variable is the monthly occurrences of the Influenza-like Illness (ILI) symptomatic cases as stated by the survey respondents and ‘Survey_Influenza’ variable is the monthly occurrences of the seasonal influenza cases as stated by the survey respondents

	GPs_Influenza	Survey_Symptoms	Survey_Influenza
**GPs_Influenza**	1	**0.935** (*p*-value = 0.002)	**0.929** (*p*-value = 0.002)
**Survey_Symptoms**		1	**0.890** (*p*-value = 0.007)

Please note: ‘GPs_Influenza’ is the diagnosed seasonal influenza individuals collected by the GPs, while ‘Survey_Symptoms’ variable is the monthly occurrences of the Influenza-like Illness (ILI) symptomatic cases as stated by the survey respondents and ‘Survey_Influenza’ variable is the monthly occurrences of the seasonal influenza cases as stated by the survey respondents

#### Google trends

In addition, we compared both the results of the surveys and the GP notifications with the Google Trends records using the key words ‘Influenza’, ‘Flu’ and ‘Cold’ in both Maltese and English language for searches that were carried out in Malta. The Google searches (Fig. 1g, h) all show a peak in January, as seen in other data, but the dynamics in other months does not capture the rise in cases in October–December and fall in February–May periods (Fig. 1e, f).

## Discussion

In order to successfully predict and control infectious disease outbreaks and to design an appropriate public health response to an unfolding epidemic, we need to be able to accurately estimate the number of cases. There are different ways to achieve this, most notably medical records provided by GPs. The official Health Authority data are often assumed to give us the best estimation of the trends and they – unlike the telephone survey – can provide the real-time estimation of an unfolding epidemic [1]. However, none of these methods can capture all infection cases and so a combination of different approaches is needed to fully understand the dynamics of the outbreak.

As demonstrated in our paper, the cross-sectional surveys carried out via telephone are easy to conduct and can include extensive information about seasonal influenza (Table 8). However, most data are based on self-diagnosis and self-reporting. Telephone surveys are subject to self-selection bias and non-representativeness of the sample. While the latter can be corrected through post-stratification techniques which are standard in survey research to approximate a representative sample of the general population, the self-selection bias is harder to address as it can depend on specific interests (e.g., in health-related topics, surveys) and specific subpopulation. Additionally, retrospective surveys suffer from recall bias effects since data collection is dependent on people’s memory about recalling past events such that respondents were asked to report their symptoms and other health indicators (e.g. body temperature, medicine uptake, and health seeking behaviour) which had occurred several months before the interview. However, such a recall bias is more likely to be minimal when individuals tend to recall health events that threaten their life’s stability. Another important issue with this type of retrospective surveys is time lag with which the data are collected and made available.

**Table 8:** Different sources of information in relation to the seasonal influenza

Source of Data	Type of Data	Advantages	Disadvantages
Telephone Surveys	- Influenza Symptoms - Number of influenza cases - Number of hospitalisation due to influenza - Respondents’ medical information - Other dynamics in relation to the influenza	- Easy to collect - Has the possibility to include extensive information about the outbreak - Data can be compared against non-influenza individuals	- Relies on self-diagnosis and self-reporting
GP reporting	- Consultations - Diagnosed	- Medical data - Weekly data	- Consultation include other cases - Average of 6 doctors out of 300 reporting cases; low coverage
Google Trends	- Search by popularity	- Quick and easy to collect	- Data relies on internet connected individuals only - Data does not tell you the severity of the outbreak
Online surveys	Not available in Malta		
Serological data	Not available in Malta		

Telephone surveys are time consuming as the interviewer needs to read out every question. Faced with the same question online, the respondents can review all questions in further detail. Online surveys and serological data in relation to the seasonal influenza are not available in Malta, but the online surveys introduce additional bias as they capture only a subsection of the population. On the other hand, telephone surveys give researchers access to individuals without the expense and time consumed by travel to different locations and the possibility to interview individuals who may not otherwise be available.

Although the reporting efficiency of ILI surveillance data can be quite low, GPs data are based on instantaneous medical expert diagnosis and hence this makes data available in real time, which in turn is passed on to Health Authorities in a timely fashion. Google Trends provide an indication about the peak of the outbreak, but it fails to provide information about the severity of the same outbreak.

Using the survey data, we were able to estimate the number of individuals in Malta who had acquired seasonal influenza during 2014–15 season as between 180,000 and 230,000. Thus, between 42 and 54% of the Maltese citizens had seasonal influenza during the 2014–15 period, while for the 2015–16 season, this varied between 135,000 (31.8%) and 200,000 (47.1%) individuals. According to the CDC, seasonal influenza in other countries such as the United States affects between 5 and 20% of the total population [10]. In Finland, for instance, it was estimated that 6% were infected during the first wave of the 2009–10 pandemic season and 3% during the second wave [46]. However, none of these estimates were based on cross-sectional surveys, but rather through on-line and national surveillance data [48]. Due to Malta being a densely populated island nation, it is difficult to directly compare Malta’s incidence rate with other countries [47, 49], as physical contact between people is more likely to occur in Malta, and so the transmission rate of seasonal influenza can be greater than that in other countries [10].

## Conclusions

There are no studies that focus on analysing in depth factors related to the quality of seasonal influenza reporting and details of the symptoms in Malta. Throughout this research study, we demonstrated that telephone surveys are a reliable way to collect epidemiological data and can be used to estimate the total number of seasonal influenza cases. They also give a unique insight into other important factors like self-perception and distribution of symptoms among the population. We have also showed that when two surveys are carried out at different times in relation to the seasonal influenza outbreak (either after the outbreak, or during the tail of the epidemic), the results from the two surveys are comparable. Thus, we claim that this novel method helps to provide another useful data set for medical statistics, in addition to those provided by the official notifications by GPs and by Google Trends. Furthermore, these sources of data can be used together to provide a more comprehensive picture of the influenza season. The collected information can be used to improve the epidemiological modelling related to the seasonal influenza and other infectious diseases, and thus can contribute to public health initiatives [49]. In particular, we used the survey data to estimate the reporting rate of the GP system that forms the basis for official records and hence underlies the key decisions taken by the Health Authorities. We showed that based on the two seasons, the reporting rate in Malta varies between 14 and 22%.

However, we need to treat such results with caution. To a certain extent, we are comparing self-diagnosis of individuals against the GPs seasonal influenza diagnosis. Hence, the baseline for both numbers is most likely not the same. The self-diagnosis provides an estimate of the actual seasonal influenza cases based on personal perception. Furthermore, it is important to note that some of the above symptoms overlap with other respiratory viruses and as a result, these estimates are mostly likely an over-estimate. Nevertheless, the correlation analyses showed that the survey results are a true representation of the dynamics and patterns of the incidence of the seasonal influenza. The monthly data between the survey and GPs data (Fig. 1) match fairly well, thus providing an extra level of confidence that the respondents were accurately remembering their medical history for the past year. Participation and response rates in epidemiological surveys are very important [50], therefore the right methodologies are needed to ensure that the response rate is satisfactory. Such studies can already contain certain elements of bias, since several responses are based on the respondents’ medical knowledge. For a noticeable number of questions, respondents often base their judgement on self-medical diagnosis. This is considered an important element in epidemiological studies as it supports pandemic control strategies through self-management practices and the reduction of visits to healthcare facilities, thereby aiding to contain viral spread [51]. Self-reports have been compared to patients’ electronic medical records [48] in order to examine the accuracy of self-reporting vaccination status. Furthermore, there is substantial evidence about the accuracy of self-reports of seasonal influenza, particularly during pandemics [40, 52–58], showing the benefits of self-reported symptoms to complement traditional surveillance data.

Most of the results related to the symptoms are in accordance to the findings of the UK flu survey [32], which reports the most common symptoms as runny nose, cough, sneezing, headache, sore throat and feeling tired. However, the UK survey data are biased towards those individuals that have frequent internet access and so can skew the results towards those with a higher level of education [32].

Since GPs data was required for comparison with survey data, we assumed that the results are also representative of the whole population of Malta. The authors acknowledge this as a limitation because children often have higher prevalence of ILI/influenza [21]. Future research merits further analysis of data including those below 18 years in Malta.

Further work is warranted to understand to what extent these surveys can contribute to our understanding of disease diagnosis if they were to be conducted during an actual outbreak. Running a series of cross-sectional surveys during various stages of the seasonal influenza outbreak might provide further understanding of people’s perceptions of seasonal influenza, and probe deeper into whether the survey results are time-dependent. Furthermore, scientific surveys can provide detailed information to understand the real notion of seasonal influenza, and to offer an opportunity to improve the prior information for future epidemiological modelling and provide even further refinements beyond this analysis. Furthermore, survey findings can be tested using other observed datasets to examine their validity in the context of epidemiological studies. All this information can aid in designing a package of different sources of information in support to the prediction of current and future influenza outbreaks.

## Supplementary Information

**Additional file 1.** )

**Additional file 2.**

## Data Availability

Data supporting the conclusions of this study are included within the manuscript. The raw datasets analysed during the current study are available from the corresponding author on reasonable request.

## Abbreviations

ARI: Acute Respiratory Infections
CDC: Centre for Disease Control and Prevention
GP: General Practitioner
ILI: Influenza-Like Illness
MHPD: Malta Health Promotion Department
NHS: National Health Service
NSO: National Statistics Office
PHE: Public Health England
SD: Standard Deviation
UK: United Kingdom
WHO: World Health Organization

## Footnotes

1 https://www.surveysystem.com/sscalc.htm

2 https://trends.google.com/trends/explore?geo=MT&q=influenza
