# Supplementary material for: Cross-sectional telephone surveys as a tool to study epidemiological factors and monitor seasonal influenza activity in Malta
Source: BMC Public Health. 2021 Oct 9;21:1828. doi: 10.1186/s12889-021-11862-x (PMC8502089; doi:10.1186/s12889-021-11862-x)
Supplement: Supplementary file 1 — Additional file 1. ) [file 12889_2021_11862_MOESM1_ESM.pdf]

Under reporting Seasonal Flu Survey (English Version)

Gender _____ Locality ________________ Age _____ Married _______
Status: Employee □ Student □ Housewife □ Unemployed □ Pensioner □
What is your job?________________________________ Level of Education reached __________
Number of individuals at your household (including you): ________ What is their age? _______

1. What is your main means of transport?
   Walking ___ Bike ___ Motorbike ___ Car ___ Public transport ___ Other ___
2. Did you receive a flu vaccine this winter/autumn season? (2014-2015)
   Yes ___ No ___ I don't know ___
3. If 'No', why? ____
4. How many times did you visit your GP (doctor) during this past year? ________________
5. Do you take regular medication for any medical conditions such as asthma, diabetes, heart disorders, kidney disorder or other? Yes ___ No ___ I don't know ___
6. Do you smoke? Yes ___ No ___
7. If 'Yes', how many cigarettes per day? ________
8. Have you had any of the following symptoms during the past year?
   Fever ___ runny or blocked nose ___ Sneezing ___ Sore throat ___ Cough ___
   Shortness of breath ___ Headache ___ Muscle/joint pain ___ Chest pain ____
   Feeling tired or exhausted ___ Loss of appetite ___ Watery eyes ___ Nausea ___
   Vomiting ___ Diarrhoea ___ Stomach ache ___ Other symptoms ___ Nothing ___
9. When did your symptoms appear for the above during the past year? ___________
10. Were you restricted to staying at home? Yes ___ No ___ I don't know ___
11. Approximately, in days, how long was the duration for the above symptoms? _________
12. Since 'August 2014' did you have the seasonal influenza? Yes ___ No ___ I don't know ___
13. If 'Yes', when did you have the seasonal influenza? ___________________
14. Approximately, in days, how long was the duration of influence? ____________________
15. What were the symptoms? ____
16. Did you have high temperature? Yes ___ No ___ I don't know ___
17. Because of your seasonal influenza fever, did you visit a doctor?
    Yes ___ No ___ I don't know ___
18. Did you take any medication due to this influenza? Yes ___ No ___ I don't know ___
19. Were you hospitalized due to your influenza? Yes ___ No ___
20. If 'Yes' for how many nights? __________
21. Did any of your household members had the seasonal influenza?
    Yes ___ No ___ I don't know ___
22. If 'Yes', how many members? ________
23. And what is their age? _______________
