# Supplementary material for: Cross-sectional telephone surveys as a tool to study epidemiological factors and monitor seasonal influenza activity in Malta
Source: BMC Public Health. 2021 Oct 9;21:1828. doi: 10.1186/s12889-021-11862-x (PMC8502089; doi:10.1186/s12889-021-11862-x)
Supplement: Supplementary file 2 — Additional file 2. [file 12889_2021_11862_MOESM2_ESM.docx]

**Appendix - Survey 2014/2015 results**


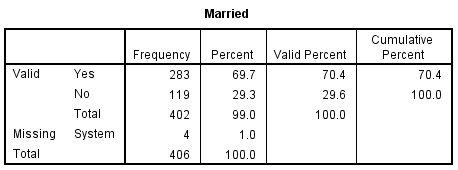


**Table 1 – Respondents’ marital status**


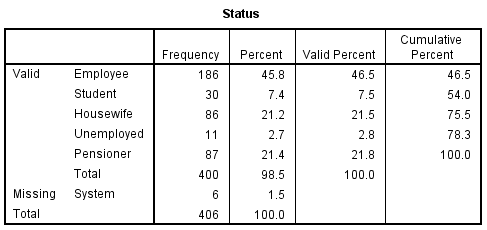


**Table 2 – Respondents’ occupational status**


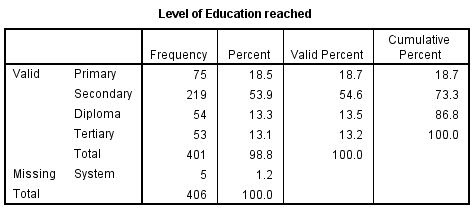


**Table 3 – Respondents’ level of education**


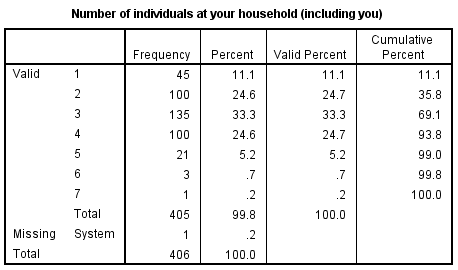


**Table 4 – Respondents’ number of individuals in their household**


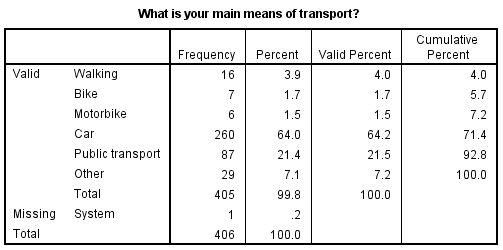


**Table 5 – Respondents’ main means of transport**


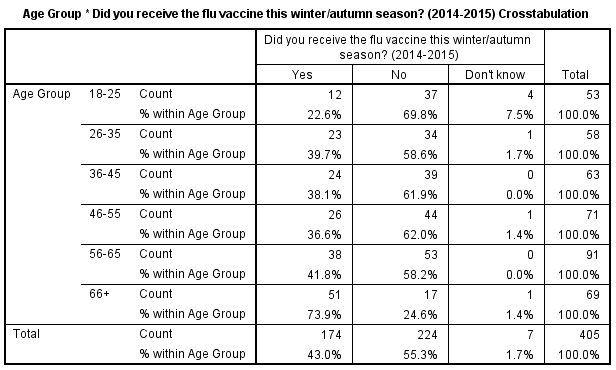


**Table 6 – Respondents’ flu vaccine uptake compared with their respective age group.**


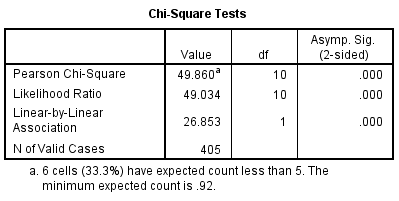


**Table 7 – Chi-Square test of association between flu vaccine uptakes compared with their respective age group.**


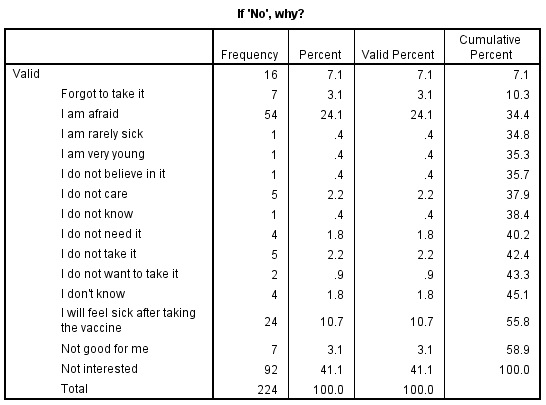


**Table 8 – Respondents’ reasons for not taking the flu vaccine.**


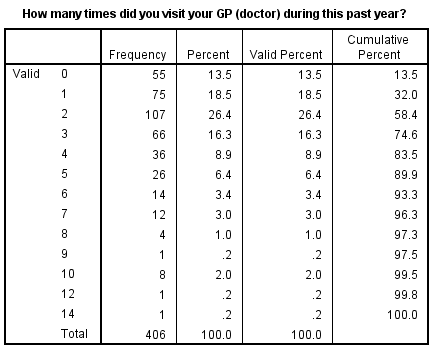


**Table 9 – The number of times respondents visit their GP throughout the whole year.**


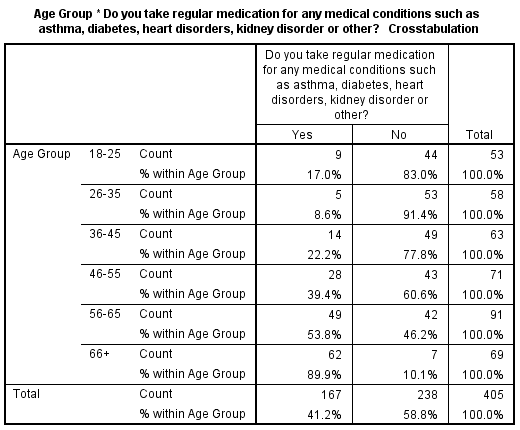


**Table 10 – Respondents’ frequency of regular medication compared with their respective age group.**


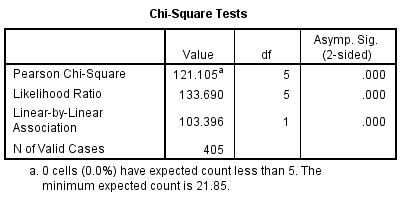


**Table 11 – Chi-Square test of association between the frequencies of regular medication compared with their respective age group.**


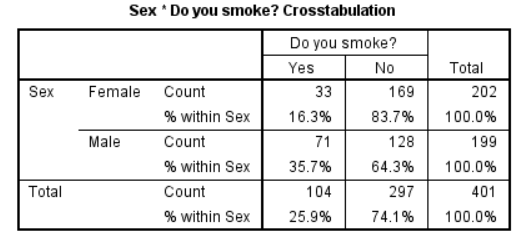


**Table 12 – Cross tabulation between the frequencies of smokers compared with sex.**


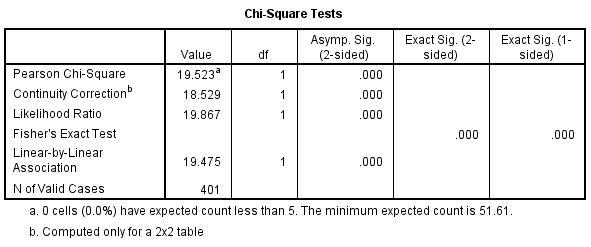


**Table 13 – Chi-Square test of association between the frequencies of smokers compared with sex.**


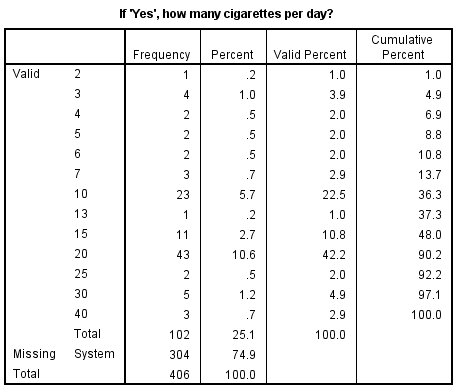


**Table 14 – The number of cigarettes respondents consume per day.**


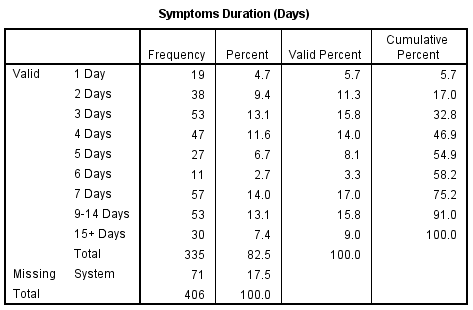


**Table 15 – The number of days for the influenza-like-illness symptoms to persist according to survey respondents.**


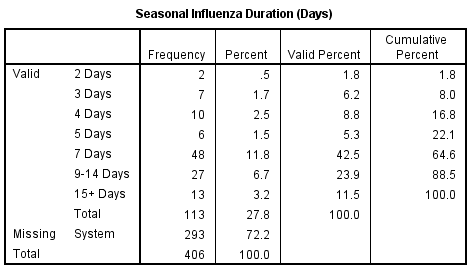


**Table 16 – The number of days for the seasonal influenza to persist according to survey respondents.**

**Nights at hospital due to the seasonal influenza:**
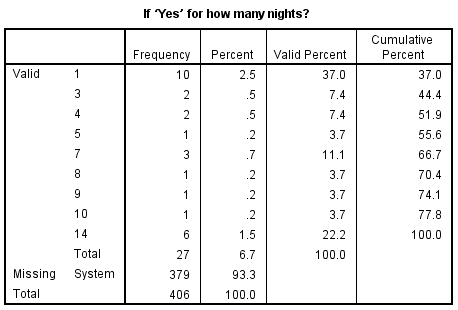


**Table 17 – The number of days of hospitalisation due to seasonal influenza.**


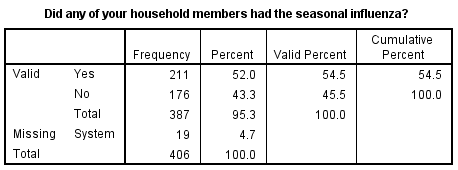


**Table 18 – Household members that had acquired the seasonal influenza.**


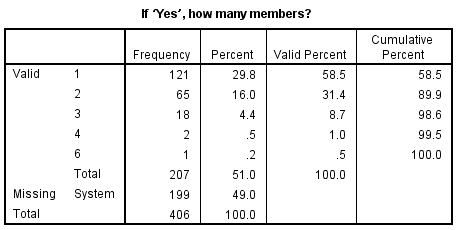


**Table 19 – The number of members within the respondents’ household that had acquired the seasonal influenza.**
